# A Novel Antimicrobial Peptide (Kassinatuerin-3) Isolated from the Skin Secretion of the African Frog, *Kassina senegalensis*

**DOI:** 10.3390/biology9070148

**Published:** 2020-07-02

**Authors:** Hui Wang, Haoyang He, Xiaoling Chen, Mei Zhou, Minjie Wei, Xinping Xi, Chengbang Ma, Qiang Du, Tianbao Chen, Chris Shaw, Lei Wang

**Affiliations:** 1 School of Pharmacy, China Medical University, Shenyang 110001, China; hwang@cmu.edu.cn (H.W.); mjwei@hotmail.com (M.W.); 2 Natural Drug Discovery Group, School of Pharmacy, Queen’s University Belfast, Belfast BT9 7BL, Northern Ireland, UK; hhe06@qub.ac.uk (H.H.); x.chen@qub.ac.uk (X.C.); m.zhou@qub.ac.uk (M.Z.); x.xi@qub.ac.uk (X.X.); t.chen@qub.ac.uk (T.C.); chris.shaw@qub.ac.uk (C.S.); l.wang@qub.ac.uk (L.W.)

**Keywords:** amphibian skin secretion, *Kassina senegalensis*, antimicrobial peptide, antibiofilm, molecular cloning

## Abstract

Amphibian skin secretions are remarkable sources of novel bioactive peptides. Among these, antimicrobial peptides have demonstrated an outstanding efficacy in killing microorganisms via a general membranolytic mechanism, which may offer the prospect of solving specific target-driven antibiotic resistance. Here, the discovery of a novel defensive peptide is described from the skin secretion of the African frog, *Kassina senegalensis.* Named kassinatuerin-3, it was identified through a combination of “shot-gun” cloning and MS/MS fragmentation sequencing. Subsequently, a synthetic replicate was subjected to biofunctional evaluation. The results indicated that kassinatuerin-3 possessed antimicrobial activity against Gram-positive bacteria but no effect against Gram-negative bacteria. Additionally, it was active in biofilm eradication on *S. aureus* and MRSA and in the antiproliferation of selected cancer cell lines. Moreover, it had a very mild hemolytic effect, which demonstrated a high therapeutic index for kassinatuerin-3. Collectively, although kassinatuerin-3 did not demonstrate remarkable bioactivities compared with other natural or synthetic antimicrobial peptides (AMPs), it offered a new insight into the design of antimicrobial derivatives.

## 1. Introduction

In the past several decades, the increasing problem of antimicrobial resistance to conventional antibiotics has made the discovery and development of novel antimicrobial agents increasingly important [1,2]. The overuse, misuse, and abuse of antibiotics have led to the selection and spread of numerous resistant pathogenic microorganisms [3], and, among these, so-called ESKAPE pathogens (*Enterococcus faecium, Staphylococcus aureus, Klebsiella pneumoniae, Acinetobacter baumannii, Pseudomonas aeruginosa*, and *Enterobacter* spp.) are most commonly associated with resistance in hospitals [4]. Furthermore, these microbes can readily form biofilms which can lead to recalcitrant biofilm-related septic complications [5].

Antimicrobial peptides (AMPs) from different sources are now well-recognized as a potential solution to resistance problems due to their different mechanism of action against highly conserved membrane structures [6,7] and their antibiofilm effects [8]. Among these sources, such as single-celled microorganisms, insects and other invertebrates, plants, amphibians, birds, fish, and mammals, the AMPs from amphibian skin secretions are the most diverse in structure and have been studied for decades [9].

To date, thousands of AMPs have been discovered in amphibian skin secretions, and these peptides exhibit a high degree of amino acid sequence diversity and peptide chain lengths ranging from 10 to 50 residues [10]. Based on their structural characteristics, amphibian skin-derived AMPs have been classified into different superfamilies, such as the brevinin and temporin superfamilies from *Ranidae* frogs [11]; dermaseptin, phylloxin and phylloseptin from *Hylidae* frogs [12]; bombinin and bombinin H from *Bombinatoridae* toads [13]; and magainin from the *Pipidae* species [14]. Most of these are generally cationic and amphipathic, which helps them to interact with and disrupt lipid membranes [15,16]. Interestingly, AMPs have also shown remarkable potential in clinical trials due to their versatile biological activities. So far, several AMPs have been developed to the preclinical stage and some even to clinical trials. Pexiganan, an analogue of magainin from *Xenopus* skin, has been evaluated in two phase III clinical trials for bacterial infections of diabetic foot ulcers [17,18,19].

Although the primary structure of skin defensive peptides exhibits a high degree of variety, their cDNA encoding biosynthetic precursors still retains similar topological structures, consisting of a signal peptide domain, a spacer peptide domain, and a mature bioactive peptide domain at the C-terminus following a typical prohormone processing signal, Lys-Arg [20]. The former two domains are highly conserved among the bioactive peptide precursors from closely related species, while the latter shows remarkable differences in its primary structure. The bioactive peptide can be released from the precursor by the site-specific cleavage of endoproteolytic enzymes in the skin secretion [21]. Most of these enzymes are dipeptidyl peptidases which recognize the basic or hydrophobic residues at specific sites and typically remove the signal peptide or spacer peptide domains [22]. Additionally, most skin defensive peptides possess post-translational modifications that play a vital role in ensuring their biological efficacy and potency. For instance, the C-terminal amide is produced from a Gly residue amide donor at the C-terminal end of the precursor, by the action of peptidylglycine amidating monooxygenase [23]; the loop conformation (the so-called ranabox motif) is formed by a disulphide bridge between two Cys residues in the sequences of brevinin peptides [24] and ranacyclin peptides [25].

*Kassina* is a unique genus of African frog, a species which is famed for their ability to walk/run instead of jumping or swimming. Their copious white and sticky skin secretions also demonstrate a special defense strategy for their survival. Since 1977, the discovery of tachykinin peptides [26], smooth muscle myotropic peptides [27], histidine-releasing peptide [28], and AMPs [29,30] has demonstrated that their skin secretions are a promising pool for isolating bioactive peptides [26]. Although the mature peptides show a high degree of variation in structure, their cDNA-encoded biosynthetic precursors contain highly conserved signal peptide domains, hydrophilic spacer peptide regions rich in basic and acidic amino acid residues, and a common C-terminal amide donor Gly residue [29,31,32].

Among these bioactive peptides, there have only been eight AMP structures from species of this genus deposited in the Uniprot database (Access time 14 May 2020), which include one kassinatuerin-1 peptide [30], five kassinatuerin-2 peptides [29], one kassorin S [32], and one senegalin peptide [31]. Kassinatuerin-1 (GFMKYIGPLIPHAVKAISDLI-NH_2_) showed broad-spectrum antimicrobial activity with relatively high cytolytic activity against mammalian cells [33]. Kassinatuerin-2 from *K. senegalensis* (FIQYLAPLIPHAVKAISDLI-NH_2_) was devoid of antimicrobial activity against the standard tested microorganisms [30], while the other four from *K. maculata* revealed weak antimicrobial activity [29]. Additionally, kassorin S and senegalin have been reported from the skin of *K. senegalensis* as shorter peptides with antimicrobial activity. Overall, the number of AMPs known to be present in the skin secretions of species of the *Kassina* genus is still limited when compared to those found in *Ranidae* and *Hylidae* species. It also suggests that these *Kassina* frogs have a high potential for the discovery of novel AMPs and possibly other unique peptides as well. 

Here, we report the isolation, structural characterization, functional profiling, and nucleotide sequence of the precursor cDNA of a novel AMP, named kassiniatuerin-3, from the defensive skin secretion of the African frog, *K. senegalensis*, with the following primary structure: FIQHLIPLIPHAIQGIKDIF-NH_2_.

## 2. Materials and Methods

### 2.1. Specimen Biodata and Secretion Acquisition

Skin secretion acquisition from the frog, *K. senegalensis,* has previously been described in detail [34]. Briefly, the frogs were stimulated by a mild battery current on the dorsal skin surface to discharge the defensive skin secretion from the granular glands. This was rinsed from the skin using distilled deionized water and collected into a chilled beaker. This was then lyophilized and stored at −20 °C before use. The study was performed according to the guidelines of the UK Animal (Scientific Procedures) Act of 1986, under project license PPL 2694 (M.Z.), issued by the Department of Health, Social Services and Public Safety, Northern Ireland. Procedures had been vetted by the Institutional Animal Care and Use Committee (IACUC) of Queen’s University Belfast and approved on 1 March, 2011.

### 2.2. Molecular Cloning of Kassinatuerin-3 Precursor-Encoding cDNA

The molecular cloning method that was employed has been described in detail before [35]. Five mg of lyophilized skin secretion was dissolved in 1 mL of cell lysis/mRNA protection buffer obtained from Dynal Biotech, Wirral, UK. Polyadenylated mRNA was isolated from this by using magnetic oligo-dT Dynabeads, as described by the manufacturer (Dynal Biotech, Wirral, UK). The isolated mRNA was then subjected to 3′-rapid amplification of cDNA ends (RACE) procedures to obtain full-length kassinatuerin-3 precursor nucleic acid sequence data using a SMART-RACE kit (Clontech, Oxford, UK), likewise as per the manufacturer’s instructions. Briefly, the 3′-RACE reactions employed a nested universal (NUP) primer (supplied with the kit) and a degenerate sense primer (KS; 5′- GCCATGARGACCYTCATTCTGCT-3′) that was designed to a highly-conserved domain of the 5′-untranslated region of previously characterized cDNA sequences from the *Kassina* species. The 3′-RACE reactions were purified and cloned using a pGEM-T vector system (Promega Corporation) and sequenced using an ABI 3100 automated sequencer.

### 2.3. Isolation of Kassinatuerin-3 from the Skin Secretion

The isolation and identification of mature kassinatuerin-3 from the skin secretion via RP–HPLC and LC–MS analyses, which were performed as in a previous study [36]. A volume of 5 mg of lyophilized skin secretion was dissolved in 1.5 mL of 0.05/99.5 (*v*/*v*) trifluoroacetic acid (TFA)/water. A volume of 1 mL of supernatant was subjected to the RP–HPLC system, fitted with a semi-preparative column (Jupiter C-18, 5 μm particle, 300 Å pore, 250 × 10 mm, Phenomenex, Macclesfield, UK), using a gradient from 0.05/99.5 (*v*/*v*) TFA/water to 0.05/19.95/80.0 (*v*/*v*/*v*) TFA/water/acetonitrile in 240 min, at a flow rate of 1 mL/min. The fractions were collected at 1 min intervals. Another 0.2 mL was injected into the LCQ fleet ion trap LC–MS, fitted with a column (Jupiter C-18, 5 μm particle, 300 Å pore, 150 × 4.6 mm, Phenomenex, Macclesfield, UK), using the same gradient. The most intensive ion in the full MS scan was subjected to MS/MS fragmentation by the normalized collision energy (NCE) of 27. The electrospray voltage was applied as 4.5 kV and the temperature of the capillary tube was set as 275 °C. The MS/MS spectrum was analyzed by Thermo Scientific Proteome Discoverer 1.0 software via the Sequest algorithm, against a self-defined FASTA database (Thermo Fisher Scientific, San Jose, CA, USA).

### 2.4. Solid-Phase Peptide Synthesis

The peptide was chemically synthesized using an automatic Tribute peptide synthesizer, as previously described [37]. The Fmoc group of amino acids was removed by 20/80 (*v*/*v*) piperidine/N, N-dimethylformamide (DMF) solution. The peptide bond was coupled with 2- (1H-benzotriazole) -1, 1, 3, 3-tetramethyluronium (HBTU) in 1 M 4-methylmorpholine (NMM)/DMF solution. Once the peptide chain was complete, it was removed from the resin using a cleavage solution containing 94% TFA, 2% water, 2% thioanisole, and and 2% 1, 2-ethanedithiol. Purification and identification of kassinatuerin-3 were achieved by RP-HPLC and MALDI-TOF mass spectrometry, respectively, following lyophilization.

### 2.5. Circular Dichorism

The secondary structure of the purified kassinatuerin-3 was analyzed by a JASCO J815 circular dichroism (CD) spectrometer, as done previously [37]. The peptide was prepared in 10 mM NH_4_AC and 50% (*v*/*v*) trifluoroethanol (TFE)/10 mM NH_4_AC solutions at a concentration of 50 µM. The pH of both buffers was adjusted to 7.4. The Heliquest tool and I-TASSER were employed to predict the helical wheel of the peptides as well as the 3D conformation.

### 2.6. Antimicrobial Assays

The antimicrobial assays were performed to detect and quantify the possible antimicrobial activities of the novel peptide. The minimal inhibitory concentration (MIC) and minimal bactericidal concentration (MBC) of kassinatuerin-3 were determined against different microorganisms which have been described before [37]. The reference strains employed in these studies included the Gram-positive bacteria, *Staphylococcus aureus* (NCTC10788), *Enterococcus faecalis* (NCTC12697), and methicillin-resistant *S. aureus* (MRSA) (NCTC12493); the Gram-negative bacteria, *Escherichia coli* (NCTC10418) and *Pseudomonas aeruginosa* (ATCC27853); and the yeast, *Candida albicans* (NCYC1467). The bacteria were cultured in Mueller–Hinton broth (MHB) and *C. albicans* was cultured in yeast extract-peptone-dextrose (YPD) medium. The microorganism cultures (5 × 10^5^ colony forming units (CFU)/mL) were treated with peptide solutions at final concentrations ranging from 512 µM to 1 μM in two-fold dilution in a 96-well plate. The absorbance of each well was measured at 550 nm, following incubation for 20 h at 37 °C. The microorganisms were also incubated with a well-known but highly hemolytic AMP, honeybee melittin, which was used as a positive control. Phosphate-buffered saline (PBS) was employed as a negative control. Experiments were repeated four times in five replicates.

### 2.7. Hemolysis Assay

The hemolysis assay was carried out by using a 2% horse erythrocyte suspension prepared from freshly defibrinated horse blood (TCS Biosciences, Buckingham, UK), which was described in a previous study [38]. Briefly, the erythrocytes were washed with phosphate-buffered saline (PBS). The peptide solutions were co-incubated with pre-washed horse erythrocytes at a range of peptide concentrations from 5 µM to 160 µM, made by two-fold dilution from stock. After a 2h incubation period at 37 °C, the supernatant of each concentration was collected by centrifugation at 1000× *g*. The absorbance of the supernatant was assessed at 570 nm by a plate reader. The PBS and 0.1% Triton X-100-treated erythrocytes were employed as negative and positive controls, respectively. Experiments were repeated three times in five replicates.

### 2.8. Anti-Biofilm Assay

The assays for the determination of minimal biofilm inhibitory concentration (MBIC) and minimal biofilm eradication concentration (MBEC) were performed as previously described [39], with the same microorganisms as described in the antimicrobial assay. Gram-positive bacteria, Gram-negative bacteria, and *C. albicans* were cultured in tryptic soy broth (TSB), Luria–Bertani (LB) broth, and RPMI-1640, respectively. For the MBIC assay, a suspension of broth-diluted bacterial culture (5 × 10^5^ CFU/mL) was incubated with the peptide solutions at 37 °C for 24 h. For the MBEC assay, 100 μL of inoculum culture was seeded onto a 96-well flat-bottom plate and incubated at 37 °C for 48 h to produce a bacterial biofilm. Afterwards, the MBEC plate was washed using sterile phosphate-buffered saline (PBS; Sigma-Aldrich, Gillingham, UK) twice and treated with peptide solutions at 37 °C for 24 h. Then, both plates for the MBIC and MBEC assays were washed with PBS and stained using 100 μL 0.1% crystal violet solution (Sigma-Aldrich, Gillingham, UK), and they were further treated with 30% acetic acid (Sigma-Aldrich, Gillingham, UK). The absorbance of each well was recorded by a Synergy HT plate reader (Biotech, Minneapolis, MN, USA) at 595 nm. Again, melittin and PBS were used as positive and negative controls, respectively. Experiments were repeated four times in five replicates.

### 2.9. Cell Cytotoxicity

The MTT assay was carried out to determine the cytotoxicity of the novel peptide, and this assay was the same as that described in a previous study [33]. The assays used different cell lines. NCI-H23 (ATCC^®^ CRL-5800), NCI-H157 (ATCC^®^ CRL-5802), NCI-H460 (ATCC^®^ HTB-177), and NCI-H838 (ATCC^®^ CRL-5844) cells were derived from non-small cell lung cancers. LNCaP (ATCC^®^ CRL-1740™) was derived from human prostate cancer and U-251 MG (09063001) was derived from glioblastoma (astrocytoma). Briefly, 5000 cells were seeded into separate wells of a 96-well plate and incubated overnight. The peptide solutions were prepared from 10^−4^ to 10^−9^ M by 10-fold dilution in a serum-free medium and added to replace the culture medium in the 96-well plate. PBS was used as a negative control, and paclitaxel (1 μM) was used as a positive control to compare the anti-proliferative effect of the peptide. A 10 μL volume of MTT (5 mg/mL) was added to each sample after 24 h of treatment and continually incubated for 4 h. Then, the formazan crystal in each well was dissolved by dimethyl sulphoxide (DMSO) and the absorbance of the samples was measured by a Synergy HT plate reader (Biotech, Minneapolis, MN, USA). Experiments were repeated three times in five replicates.

## 3. Results

### 3.1. Molecular Cloning of Kassinatuerin-3 Peptide Precursor Transcript

**Figure 1:**
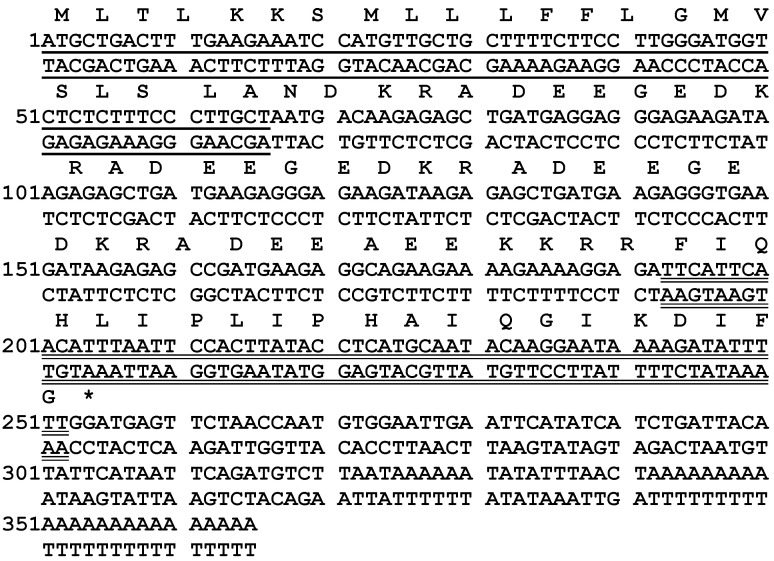
Nucleotide and translated open-reading frame amino acid sequence of the biosynthetic precursor-encoding DNA of kassinatuerin-3. The putative signal peptide sequence is single-underlined, and the mature peptide sequence is double-underlined. The stop codon is indicated by an asterisk (*).

**Figure 2:**
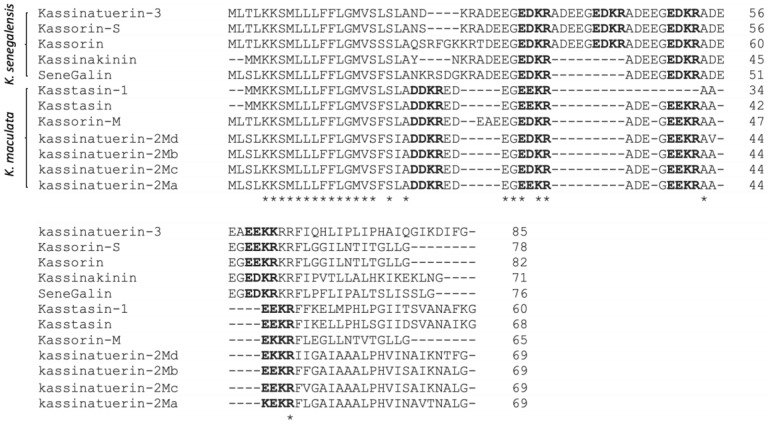
The alignment of the amino acid sequences of the biosynthetic precursors of bioactive peptides discovered in the skin secretions of *K. senegalensis* and *K. maculata*. Identical amino acids are indicated by asterisks (*). The acidic/basic tetrapeptide motif is indicated in bold typeface.

One peptide-encoding precursor was successfully and repeatedly cloned by “shot-gun” cloning (12 identical clones) from the skin secretion cDNA library of the African frog, *K. senegalensis* (Figure 1). The biosynthetic precursor open-reading frame consisted of 84 amino acid residues. The putative signal peptide domain was determined by NCBI-BLAST, and it was constituted by the first 22 amino acid residues. Following this, there is a hydrophilic domain, also known as the acidic amino acid-rich spacer peptide domain, where a large proportion of amino acid residues are acidic. The alignments of the amino acid sequences of the peptide precursors identified from *K. senegalensis* and *K. maculata* demonstrated the high degree of primary structural conservation among the signal peptide and spacer domains (Figure 2). The structural motif of -DDKR- (where D can be replaced by E) was repeated in the spacer peptide. The putative mature peptide was revealed at the C-terminal region, consisting of 20 amino acid residues. The Gly at the end acted typically as a C-terminal amide donor for the mature peptide. The putative mature peptide demonstrated the same dipeptide, -PH-, as kassinatuerin-2 in the middle of the peptide sequence. However, the remainder of the peptide exhibited limited further similarity. Therefore, the novel peptide was named kassinatuerin-3. The nucleotide sequence of the cloned precursor-encoding cDNA has been deposited in EMBL under the accession number HG794245.

### 3.2. Identification and Structural Analysis of Kassinatuerin-3

**Figure 3:**
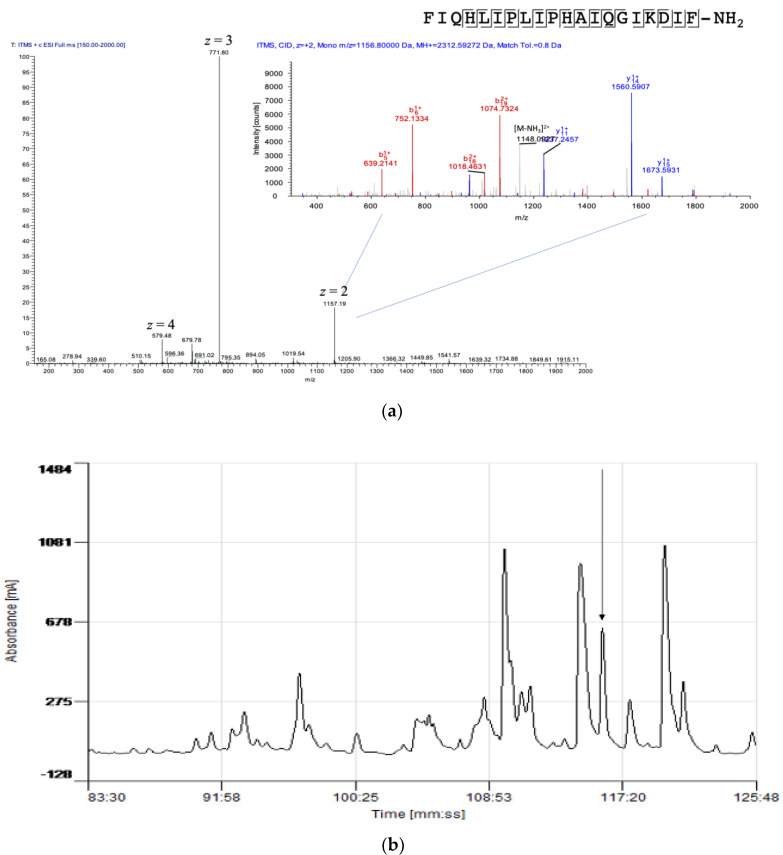
(**a**) Electrospray ion-trap MS/MS fragmentation analysis of kassinatuerin-3 in the skin secretion. The full mass scan exhibited multiple positively charged ions of kassinatuerin-3. The doubly charged precursor ion was subjected to MS/MS fragmentation (in the upper right-hand corner). The observed singly and doubly charged b-ion and y-ion fragments m/z ratios are colored as red and blue, respectively. (Note that, in the sequence call, I/L residues are isobaric and cannot be differentiated here. The assignation of I/L is done by reference to the cloned precursor template.) (**b**) Region of RP–HPLC chromatogram of the skin secretion of *K. senegalensis*, with an arrow indicating elution/retention time of the fraction (#116) containing the antimicrobial peptide, kassinatuerin-3. The *Ɣ*-axis indicates milli-absorbance units at *λ* = 214nm.

To determine the presence of kassinatuerin-3 in the skin secretion of *K. senegalensis*, the dissolved skin secretion was subjected to RP–HPLC and LC–MS/MS analysis. The MS/MS spectra were searched against the cDNA precursor of kassinatuerin-3 and this proved the presence of kassinatuerin-3 in the skin secretion as well as the C-terminal amidation of the mature peptide (Figure 3a). The observed molecular mass of kassinatuerin-3 was 2311.59 Da, which was consistent with that of the predicted mature peptide (2311.346 Da (mono.)) from the cDNA precursor. In the RP–HPLC chromatogram, the retention time of kassinatuerin-3 was around 116 min (Figure 3b). The fraction of the single peak indicated in the chromatogram was collected and exhibited antimicrobial activity via a screening test (data not shown). Therefore, we decided to chemically synthesize the peptide for further investigation.

### 3.3. Prediction of Secondary Structure and Physiochemical Properties

**Figure 4:**
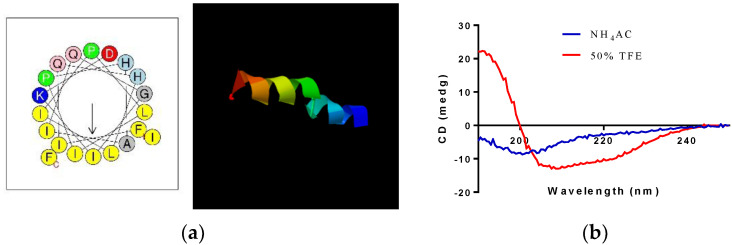
(**a**) Helical wheel diagram and 3D model of kassinatuerin-3 predicted by I-TASSER. (**b**) Circular dichroism (CD) spectra of 50 µM kassinatuerin-3 in aqueous solution (10 mM NH_4_AC) and membrane-mimicking solution (50% (*v*/*v*) trifluoroethanol (TFE)/10 mM NH_4_AC).

Kassinatuerin-3 was successfully synthesized and purified via RP–HPLC (Figure S1). The retention time and the MS/MS profile are consistent with the nature peptide in the skin secretion (Figures S1 and S2). A helical wheel diagram and a 3D model of kassinatuerin-3 are shown in Figure 4. The physiochemical properties of kassinatuerin-3 are shown in Table 1 and indicate that kassinatuerin-3 contains a net charge of +1 and high hydrophobicity. Prediction of the peptide secondary structure by CD (Figure 4b) revealed a random coil in the aqueous solution and an adopted helical structure in the 50% TFE solution.

### 3.4. Antimicrobial and Hemolysis Assays

A synthetic replicate of the novel peptide was tested for antimicrobial activity against the Gram-positive bacteria, *S. aureus*, methicillin-resistant *Staphylococcus aureus* (MRSA), and *E. faecalis*; the Gram-negative bacteria, *E. coli* and *P. aeruginosa*; and the yeast, *C. albicans*. The MICs and MBCs of kassinatuerin-3 against several microorganisms are shown in Table 2. The peptide exhibited antimicrobial activity against the three Gram-positive bacteria and the yeast but was ineffective against the Gram-negative bacteria at the highest concentration tested.

### 3.5. Antibiofilm Assay

The antibiofilm activities of kassinatuerin-3 were determined as minimum biofilm inhibitory concentrations (MBICs) and minimum biofilm eradication concentrations (MBECs). Kassinatuerin-3 exhibited antibiofilm activity on Gram-positive bacteria, but it did not inhibit or eradicate the biofilm of the Gram-negative bacteria selected for this study (Table 3). Moreover, it only inhibited the formation of biofilm in *C. albicans* but did not eradicate the mature biofilm at concentrations up to 512 µM.

### 3.6. Hemolytic and Antiproliferative Activity

**Figure 5:**
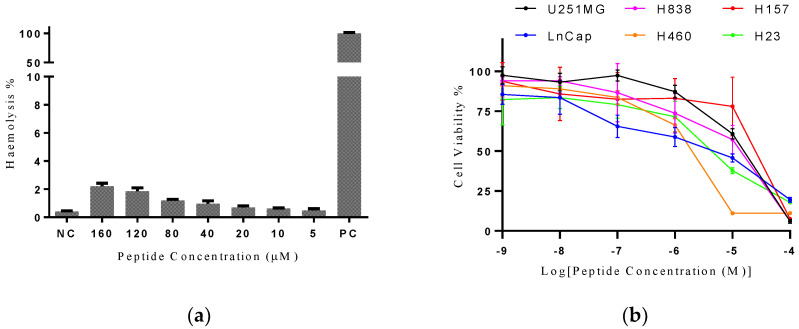
(**a**) Hemolytic activity of kassinatuerin-3 on the horse erythrocytes. PBS and 0.1% TritonX-100 were used as the negative control (N) and positive control (P), respectively. (**b**) Anti-proliferative effects of kassinatuerin-3 on a range of cancer cells within a concentration range of 10^−4^ to 10^−9^ M. Paclitaxel (1 μM) was used as the positive control. The 100% cell viability was measured in the cells with serum-free medium only. Data represent means ± SD of five replicates.

Kassinatuerin-3 produced no detectable hemolysis activity up to a concentration of 160 μM, above which there was slight activity (Figure 5a). The peptide was then assessed for possible anti-proliferative effects on a range of cancer cell lines. Six different cell lines were employed in the cell viability assays, with three cell lines (H23, H157, H838 and H460) representing human non-small cell lung cancers, LNCaP representing a human prostate cancer and U251MG was from a glioma. The peptide displayed significant dose-dependent anti-proliferative activity against all the cell lines employed in the study (Figure 5b), with IC50s showed in Table S1.

## 4. Discussion

The development of natural medicines came about when primitive humans found that both animals and plants were good sources [40]. Meanwhile, the establishment of bionics allowed researchers to study the structures and functions of objects in specific environments, which could model the mechanisms of several diseases [41]. Animal-based medicines, such as insulin, have played a remarkable role in the prevention and treatment of diseases and health care functions. Amphibian skin secretions are rich sources of many bioactive molecules, including antimicrobial peptides (AMPs) [42,43,44]. With the overuse of antibiotics in recent years, bacteria developing resistance to these has become a huge problem [45]. In particular, antimicrobial resistance in pathogenic Gram-positive bacteria is a major issue [46] and, with these, AMPs have shown efficacy.

*K. senegalensis,* an African frog species, has, like many frogs, been shown to express bioactive peptides in its skin secretion, and these may represent potential therapeutic agents [31,32]. However, compared with other amphibian species, such as *Phyllomedusa* and *Rana* frogs [47,48,49], the number of the identified peptides is much lower in the skin secretion of *K. senegalensis*. With regards to AMPs, only kassinatuerin-1 and -2 and senegalin have been reported [29,30,31], implying that it remains a promising resource for novel AMP discovery.

The present study has employed the integration of isolation/structural characterization and molecular cloning approaches to reveal the presence of a novel peptide, kassinatuerin-3, in the lyophilized skin secretion of *K. senegalensis*. Kassinatuerin-3 has 20 amino acids and shows some sequence identity to another AMP from this species, kassinatuerin-2, as revealed by the bioinformatic analysis of the precursor using the NCBI database. They exhibited the common structural characteristics of C-terminal amidation and internal motif -L/IPH- [29]. The biosynthetic precursor contains the highly conserved signal peptide domain, not only from the same species but also from a closely related species. The spacer peptide has an identity with the kassorin S precursor, and the specific motif, -DDKR-, was also repeated in the other precursor. These features suggest that the peptide encoding genes could have originated from a common ancestor [50]. The ancient gene could encounter a rapid mutation and development due to rapid changes in the environment and survival strategies, resulting in the rapid diversification of encoded peptides.

Kassinatuerin-3 has a remarkable therapeutic index due to its low toxicity to normal cells whilst having antimicrobial activity against Gram-positive bacteria. The MICs against *S. aureus* and MRSA were 32 µM and 64 µM, respectively. Meanwhile, kassinatuerin-3 did not lyse the membranes of red blood cells, even at a concentration as high as 160 μM, which is the maximum concentration required to kill bacterial and cancer cells. However, the overall bioactive potency of kassinatuerin-3 is moderate compared to melittin and other lead AMPs (e.g., magainin and gramicidin D [9]). In the process of antimicrobial action, electrostatic attraction plays a major role in facilitating interaction between the negatively charged components of bacterial and cancer cell membranes and the positively charged AMPs [51]. As a previous study of kassinatuerin-1 and designed analogs reported, increasing cationicity significantly improves the antimicrobial and cytolytic activity [33]. Kassinatuerin-3 possessed a +1 net charge at the physiological pH, which is not only believed to facilitate the electrostatic interaction with bacterial cell membranes but also maintains the selectivity. Kassinatuerin-1 with +2 net charges demonstrated an MIC of 6.25 μM against *S.aureus* but induced 50% hemolysis at a concentration of 65 μM. Meanwhile, kassinatuerin-2Ma, possessing the same net charge as kassinatuerin-3, exhibited a slightly better result against *S.aureus* (MIC = 16 μM) [29].

On the other hand, improving the cell selectivity of AMPs in order to increase the net charge has been reported as well [52]. These results imply that the mechanism of action may be highly specific for the primary structure of AMPs or the cellular location where AMPs are targeted as well. In our study, honeybee melittin was included as a control to monitor the effectiveness of kassinatuerin-3 as an antimicrobial agent. As expected, it was found to be very potent with all microorganisms tested, compared to kassinatuerin-3. However, despite a high potency against bacteria, this peptide is an extremely potent hemolytic and general mammalian cell cytotoxin, factors which led to its exclusion as a putative systemic antimicrobial some time ago. Moreover, more helical contents in the sequences could not only improve its antimicrobial activity but also increase its hemolytic effects. Compared to kassinatuerin-2Ma, a member of kassinatuerin-2 from *Kassina maculata*, kassinatuerin-3 reveals two proline residues, at positions 7 and 10, which could interfere with the formation of the linear helix due to the processing of proline-induced kinks in the solution. Meanwhile, that kassinatuerin-2Ma only contains one proline at conserved position 10 retained a linear helical domain at the N-terminus, which might improve the detergent-like membrane disruption effect of kassinatuerin-2Ma. It also could explain the fact that kassinatuerin-2Ma exhibited higher hemolysis (40%~50% at a concentration of 120 μM) than kassinatuerin-3 [29].

Kassinatuerin-3 showed different antimicrobial activities against Gram-positive and Gram-negative bacteria. MICs against Gram-positive bacteria were lower due to their specific membrane structures [53] and their inability to repair the disrupted bacterial membrane [54]. Additionally, the outer membrane of Gram-negative bacteria is a lipid barrier that could trap AMPs because of their hydrophobic/amphipathic nature. As a previous study of temporin-1Ta, a 13-mer AMP, found, the lipopolysaccharides (LPS) on the outer membrane could extend the peptide folding and the packing interaction between the aromatic sidechain of Phe and the hydrophobic amino acid residues in the middle of the sequences, which resulted in the formation of helical oligomeric structures [55]. This conformation could further prevent the translocation of AMPs to the cell plasma membrane, where AMPs exert antimicrobial activity. Kassinatuerin-3 contains aromatic sidechains at its N-terminus and hydrophobic residues in the middle such that it might also form oligomeric structures with LPS, resulting in the inactivity of the peptide against Gram-negative bacteria.

Previous studies indicated that AMPs have capabilities in inhibiting the growth of lung cancer cell lines [56] and some possess this anticancer function through inducing necrosis or apoptosis in these cells [57]. A number of AMPs showed immunomodulatory properties, including the activation of defense cells [58]. Moreover, the cancer cell membrane surface provides a larger potential target for AMPs due to its large surface area as caused by increased numbers of microvilli [51]. Cancer cells have low levels of cholesterol membrane anchors and hence are more sensitive to AMPs [34]. Additionally, cancer cells could possess more negative charges than normal cells due to the overexpression of glycoprotein or glycosaminoglycans on the cell membrane, which could enhance the binding effect of AMPs [59]. Interestingly, the anticancer activity of kassinatuerin-3 exhibited different levels of potency toward selected cancer cells. A possible explanation is that variations in the expression profiles of the negatively charged molecules on the cell membrane result in the changes in electrostatic force with the peptide, though we could not validate this at this stage. On the other hand, considering the low cationicity of kassinatuerin-3, the other binding approach might be associated with the presence of His residues in the sequence. A study showed that the selectivity of peptides for cancer cells can be enhanced in the acidic environment of solid tumors by the incorporation of the amino acid, histidine [60], which might suggest that kassinatuerin-3 had a cell selectivity due to its histidine content. Until now, chemotherapeutics have been widely-recognized as cancer treatments, but, in the future, AMPs which are optimized for anticancer activity could be an economically viable and therapeutically superior alternative.

## 5. Conclusions

We report the identification and biological evaluation of a novel antimicrobial peptide, kassinatuerin-3, from the skin secretion of the African frog, *K. senegalensis*. This peptide not only exhibited antimicrobial activity against selected pathogens but also inhibited the formation of biofilm in *S. aureus* and MRSA. Although kassinatuerin-3 did not exert outstanding effects compared with other AMPs, this peptide might represent a prototype peptide for the further study of the design of AMPs. Considering that there are very few AMPs which have been developed for clinical use, more comprehensive studies using experimental or longitudinal designs are clearly necessary for the development of AMPs as a lead compound of antibiotic alternatives.

## Figures and Tables

**Table 1:** Physiochemical properties of kassinatuerin-3.

Hydrophobicity <H>	Hydrophobic Moment <µH>	Net Charge z
0.880	0.570	+1

**Table 2:** Antimicrobial activity of kassinatuerin-3 against selected microorganisms. Melittin was used as a positive control.

	Kassinatuerin-3		Melittin	
	MIC ^a^ (µM)	MBC ^b^ (µM)	MIC (µM)	MBC (µM)
*S. aureus*	16–32	32–64	1–2	2–4
*E. coli*	>512	>512	1–4	8–16
*C. albicans*	64-128	128	2–8	16–32
MRSA	32–64	64–128	1–2	2–4
*E. faecalis*	128	128	1–2	4
*P. aeruginosa*	>512	>512	16–32	32–64

a. Minimal inhibitory concentration. b. Minimal bactericidal concentration.

**Table 3:** Antibiofilm effects of kassinatuerin-3 against selected microorganisms. Melittin was used as a positive control.

	Kassinatuerin-3		Melittin	
	MBIC ^a^ (µM)	MBEC ^b^ (µM)	MBIC (µM)	MBEC (µM)
*S. aureus*	16–32	64–128	1–4	16–32
*E. coli*	>512	>512	4–8	16–32
*C. albicans*	128	>512	4–16	32–64
MRSA	32–64	128	1–4	16–32
*E. faecalis*	128	256-512	4–8	16–32
*P. aeruginosa*	>512	>512	32–64	128–256

a. Minimal biofilm inhibitory concentration. b. Minimal biofilm eradication concentration.

## Supplementary Materials

The following are available online at https://www.mdpi.com/2079-7737/9/7/148/s1, Figure S1: The chromatograms of the isolated kassinatuerin-3 from the skin secretion and the purified synthetic replicates. The gradient of mobile phase is indicated by the blue line, Figure S2. MS/MS spectrum of synthetic kassinatuerin-3 that is consistent with the one of natural peptide from the skin secretion, Table S1. The calculated IC50 and SDLogIC50 of kassinatuerin-3 against each cell line. The tukey’s test for the LogIC50 of each cell line is showed below.
