# Supplementary material for: A Novel Antimicrobial Peptide (Kassinatuerin-3) Isolated from the Skin Secretion of the African Frog, Kassina senegalensis
Source: Biology (Basel). 2020 Jul 2;9(7):148. doi: 10.3390/biology9070148 (PMC7408539; doi:10.3390/biology9070148)
Supplement: Supplementary file 1 [file biology-09-00148-s001.pdf]

biology

A novel antimicrobial peptide (kassinatuerin-3) isolated from the skin secretion of the African frog, *Kassina senegalensis*

Supplementary Materials

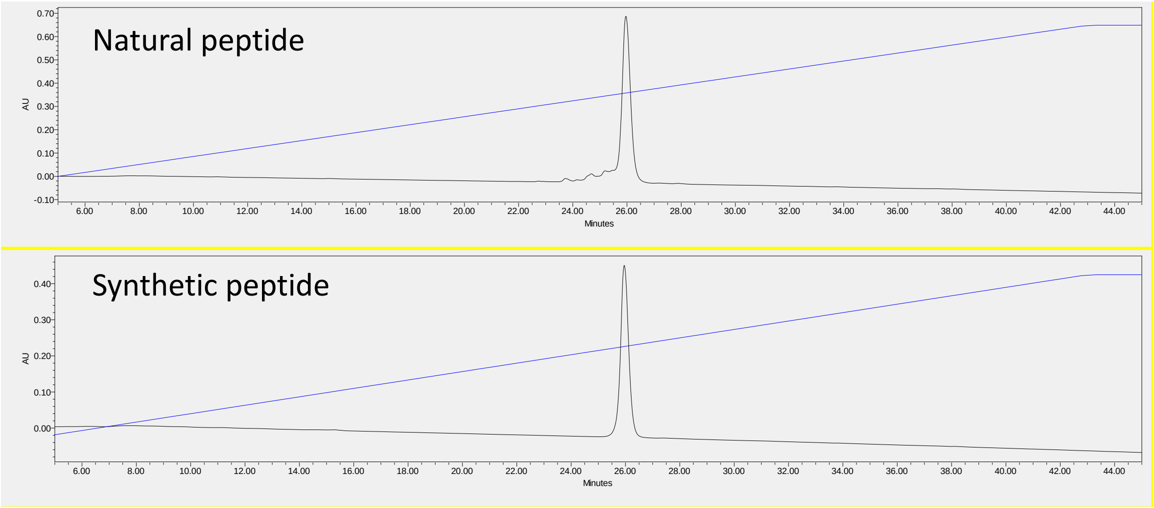

Figure S1. The chromatograms of the isolated kassinatuerin-3 from the skin secretion and the purified synthetic replicates. The gradient of mobile phase is indicated by the blue line.

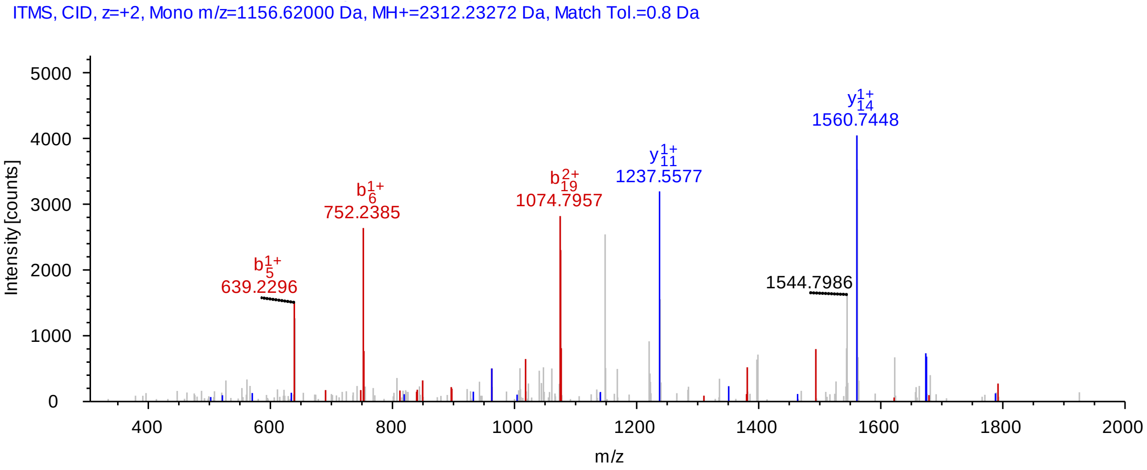

Figure S2. MS/MS spectrum of synthetic kassinatuerin-3 that is consistent with the one of natural peptide from the skin secretion.

Table S1. The calculated IC_{50} and $SD_{LogIC50}$ of kassinatuerin-3 against each cell line. The tukey's test for the LogIC50 of each cell line is showed below. The significance was indicated as $p < 0.0001$ (****), $p < 0.001$ (***), $p < 0.01$ (**), $p < 0.05$ (*) and no significance (ns).

	U251MG	LnCap	H838	H460	H157	H23
**IC_{50} (μM)**	13.20	3.79	10.03	1.67	21.54	4.52
**$SD_{LogIC50}$**	0.03648	0.1340	0.07557	0.04799	0.1050	0.09546

**Tukey's multiple comparisons test**	**Mean Diff.**	**95% CI of diff.**	**Summary**
U251MG vs. LnCap	0.541	0.470 to 0.613	****
U251MG vs. H838	0.541	0.472 to 0.610	****
U251MG vs. H460	0.541	0.476 to 0.607	****
U251MG vs. H157	−0.213	−0.282 to −0.144	****
U251MG vs. H23	0.464	0.394 to 0.535	****
LnCap vs. H838	0.0	−0.0690 to 0.0690	ns
LnCap vs. H460	0.0	−0.0657 to 0.0657	ns
LnCap vs. H157	−0.754	−0.823 to −0.685	****
LnCap vs. H23	−0.0770	−0.147 to −0.00695	*
H838 vs. H460	0.0	−0.0632 to 0.0632	ns
H838 vs. H157	−0.754	−0.821 to −0.688	****
H838 vs. H23	−0.0770	−0.145 to −0.00925	*
H460 vs. H157	−0.754	−0.817 to −0.691	****
H460 vs. H23	−0.0770	−0.141 to −0.0126	**
H157 vs. H23	0.677	0.609 to 0.745	****

© 2020 by the authors. Submitted for possible open access publication under the terms and conditions of the Creative Commons Attribution (CC BY) license (http://creativecommons.org/licenses/by/4.0/).
